# Functionally Rich Fish Assemblages Support Greater Rates of Multiple Ecological Functions in Seagrass Meadows

**DOI:** 10.1002/ece3.73011

**Published:** 2026-01-30

**Authors:** Amarina L. James, Ben L. Gilby, Andrew D. Olds, Jesse D. Mosman, Joshua T. Hill, Christopher J. Henderson

**Affiliations:** ^1^ School of Science, Technology and Engineering University of the Sunshine Coast Sippy Downs Queensland Australia; ^2^ School of Science, Technology and Engineering University of the Sunshine Coast Petrie Queensland Australia

**Keywords:** carnivory, ecosystem, fish, functional diversity, functioning, herbivory

## Abstract

Animals move between ecosystems, facilitating important ecosystem functions. The context, connectivity, and complexity of habitats alter faunal assemblages and associated ecological functions that regulate ecosystem structure, functioning, and resilience. Fish assemblages in seagrass meadows are functionally diverse and contribute essential ecological functions which are necessary for energy transfer within food webs, such as carnivory and herbivory. However, not all species support these functions equally, and it is their functional traits that modify how different species deliver ecological functions. This aim of this study was to investigate how the functional diversity of fish relates to multiple ecological functions within seagrass meadows of Moreton Bay, Queensland, Australia. This study surveyed fish community structure, using baited and unbaited remote underwater video stations, the rates of carnivory and herbivory using in‐field assays, and the composition of seagrass meadows at 50 replicates across 10 seagrass meadows in Moreton Bay, Queensland, Australia. Carnivory and herbivory increased with increasing levels of functional diversity. Seagrass cover correlated positively with functional diversity, while seagrass species diversity correlated negatively. Additionally, seagrass meadows with fewer mangroves nearby and further from reefs supported greater levels of functional diversity and, when closer to the ocean, they supported greater overall ecosystem functioning. Carnivory was greatest in seagrass meadows nearer coral reefs, while herbivory was greatest when seagrass was sparser. These findings demonstrate the importance of a functionally diverse food web and spatial context for sustaining resilient seagrass meadows and when conserving seagrass meadows in the future, understanding the functional composition of fish and seagrass will be critical. Management should prioritise the conservation and restoration of seagrass beds that are well connected with the open ocean and consider using functional diversity metrics to monitor these ecosystems given the efficacy of these metrics in reflecting broader condition and functioning.

## Graphical abstract

We show that the functional diversity of fishes promotes multifunctionality in seagrass meadows. We found that there was consistency in the seascape context and connectivity variables that structured the rate and distribution of different components of functioning and the functional diversity of fish in seagrass meadows. Finally, we found that the functional diversity of fish and the ecological functions of carnivory and herbivory correlated significantly with proximity to nearby reefs and the extent of seagrass, mangroves and coral reefs nearby.

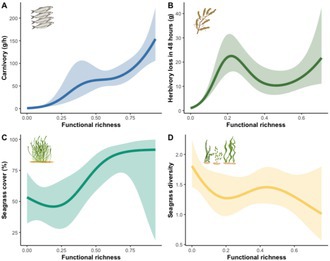

## 1 Introduction

Faunal communities and the ecosystems they reside in support a broad suite of ecological services that humans directly and indirectly derive from ecosystems (Costanza 1999; Cullen‐Unsworth and Unsworth 2013). This includes the value of animals for fisheries or harvesting and biodiversity, but also the value of ecosystems for coastal protection, sediment filtering, tourism and recreation (Cullen‐Unsworth and Unsworth 2013), with the structure and resilience of the ecosystem being partly maintained by the animal community. The species within these ecosystems support multiple ecological functions, defined as the movement, transfer and storage of energy which can occur both within and across multiple habitats or ecosystems (Estes et al. 2011; Bellwood et al. 2019). For instance, predators alter the composition of prey communities (White et al. 2003; Estes et al. 2011), herbivores regulate vegetation biomass and enhance the diversity of habitat forming species (Hyndes et al. 2014; Scott et al. 2018), and detritivores redistribute nutrients across landscapes, promoting seed dispersal and parasitic suppression (deCastro‐Arrazola et al. 2023). These ecological functions are essential for the transfer of nutrients, the structure of food webs and the resilience of habitat‐forming species (Estes et al. 2011; Biggs et al. 2020). However, the capacity for species to support these functions is drastically modified by the structure of the seascape including how well connected the seascape is, and the extent of anthropogenic pressures acting on that ecosystem (Waycott et al. 2009; Turschwell et al. 2021).

Seagrasses, as habitat‐forming species, illustrate this relationship between species, ecological functions, and anthropogenic‐induced disturbance (Campbell et al. 2018; Christianen et al. 2023). Providing important services such as nutrient cycling, carbon sequestration, food provision, and sediment stabilisation, which are further influenced by the ecological functions supported by the faunal community that inhabits seagrass meadows (Cullen‐Unsworth and Unsworth 2013; Campbell et al. 2018). Despite their importance, seagrass meadows globally are under threat and in decline (Nordlund et al. 2018; Turschwell et al. 2021). Seagrass meadows are degraded and lost due to human impacts, including pollution, destructive fishing practices, disease, invasive species, and reduced light availability related to increased sediment and nutrient runoff (Waycott et al. 2009; Turschwell et al. 2021). Consequently, seagrass meadows across the globe are, on average, less diverse, cover less extent, and have become more fragmented, reducing connectivity to surrounding coastal habitats (Waycott et al. 2009; Dunic et al. 2021). This can ultimately lead to a decline in the functioning of seagrass ecosystems (Cullen‐Unsworth and Unsworth 2013; Macreadie et al. 2014). For example, in southern Europe, the decline of seagrass meadows has led to an increase in detritivore assemblages and a decrease in herbivore assemblages (Cardoso et al. 2004). In eastern Australia, herbivorous fish play a key role in the removal of algae via consumption in seagrass meadows (White et al. 2011; Henderson et al. 2019). This function is vital for maintaining the health of seagrass ecosystems, as excessive algal growth reduces light and oxygen availability to meadows (Burkholder et al. 2007; van Tussenbroek et al. 2017). These effects are regulated by higher‐order trophic levels performing carnivory, restricting herbivore overgrazing (Wilson and Wolkovich 2011; Scott et al. 2018; White et al. 2023). Therefore, multiple functional groups throughout the food web are necessary for the maintenance of healthy seagrass ecosystems (Wilson and Wolkovich 2011; Campbell et al. 2018).

Fish are often a focal group for detecting shifts in ecological functions, serving as key indicators to these changes (French et al. 2021) because they exhibit a range of ecological, morphological and behavioural traits that drive a variation in ecological functions (Magurran et al. 2011; Villéger et al. 2017). Diverse traits among fish promote resource partitioning and foster niche overlap, enhancing diversity and increasing complex interactions such as predator–prey relationships (Wilson and Wolkovich 2011; Koutsidi et al. 2020) and the mediation of habitat‐forming species such as seagrasses through ecosystem engineering (Scott et al. 2018; Christianen et al. 2023). Fish with similar traits tend to contribute to similar ecological roles, such as food acquisition, locomotion and reproduction, thereby providing trait redundancy (McLean et al. 2019; Auber et al. 2022). Increased redundancy in trophic interactions within food webs reduces vulnerability to cascades and increases ecosystem resilience (Sanders et al. 2018; McLean et al. 2019). When fish assemblages have access to multiple habitats they exhibit greater functional richness, as connectivity between habitats provides essential food resources, shelter and refuge. Therefore, it would be expected that the impacts fish have on food webs are not equal across the seascape (Whitfield 2017; Maciel et al. 2024).

Fish migration across seascapes occurs temporally and spatially, influenced by life history stages, feeding and behavioural ecology; however, the diversity and abundance of different fish are modified by the connectivity between patches and the context those patches sit in (Fokkema et al. 2020). Greater structural complexity and connectivity between ecosystems support higher biodiversity and functioning within and across multiple habitats (Henderson et al. 2017; Maciel et al. 2024). However, when habitat connectivity becomes reduced and species movements are restricted, ecological interactions are altered (Yeager et al. 2016; Berkström et al. 2020). Fragmented seagrass meadows with low seagrass cover may support fewer species, whereas those with greater connectivity to reefs and mangroves could support a greater diversity and abundance of herbivorous fish and higher rates of herbivory (Henderson et al. 2017). Therefore, the effective management of species and ecological functions should prioritise spatial context and connectivity to multiple habitats (Olds et al. 2012; Berkström et al. 2020). Further to this, the conservation of seagrass communities and their associated fish inside marine reserves could have benefits for both the seagrass meadow cover and the fish community that resides within (Henderson et al. 2019). However, how spatial context and connectivity can modify the effect of conservation and the diversity of species, traits and multiple ecological functions across a disturbed seascape remains relatively unknown (Henderson et al. 2019).

Since seagrass ecosystems are facing significant global threats, leading to a reduction in their extent and condition (Waycott et al. 2009; Cullen‐Unsworth and Unsworth 2013), preserving the ecological functions supported by fish in seagrass meadows is critical for maintaining their resilience (Turschwell et al. 2021; Christianen et al. 2023). Studies investigating the rates of ecological functions in seagrass meadows have typically utilised simple measures of biodiversity such as abundance and species richness (Cadotte et al. 2011). While valuable, these metrics often do not adequately capture the complexity of species interactions and ecological roles (Cadotte et al. 2011; Mouillot et al. 2013). Functional diversity metrics advance on these more traditional metrics by encompassing information on the species traits of different species within an ecosystem (Schleuter et al. 2010; Cadotte et al. 2011; Mouillot et al. 2013). Understanding how the provision of multiple ecological functions varies spatially in seagrass meadows, and how these vary with changing functional diversity will be integral to the effective management of these ecosystems in the future (Henderson et al. 2019). Therefore, the aims of this study are to quantify how the functional diversity of seagrass fish communities is linked to three components of functioning in seagrass meadows: carnivory, herbivory on algae, and the condition of the seagrass meadow itself. It is expected that an increase in fish functional diversity will significantly increase functioning within seagrass meadows (Lefcheck and Duffy 2015; White et al. 2023). Further, it is expected that reserves will enhance functional diversity, multiple ecological functions and overall ecosystem functioning (Olds et al. 2012) and that seascape context and proximity would influence the distribution of these ecosystem components.

## 2 Methods

### 2.1 Study Area

**FIGURE 1:**
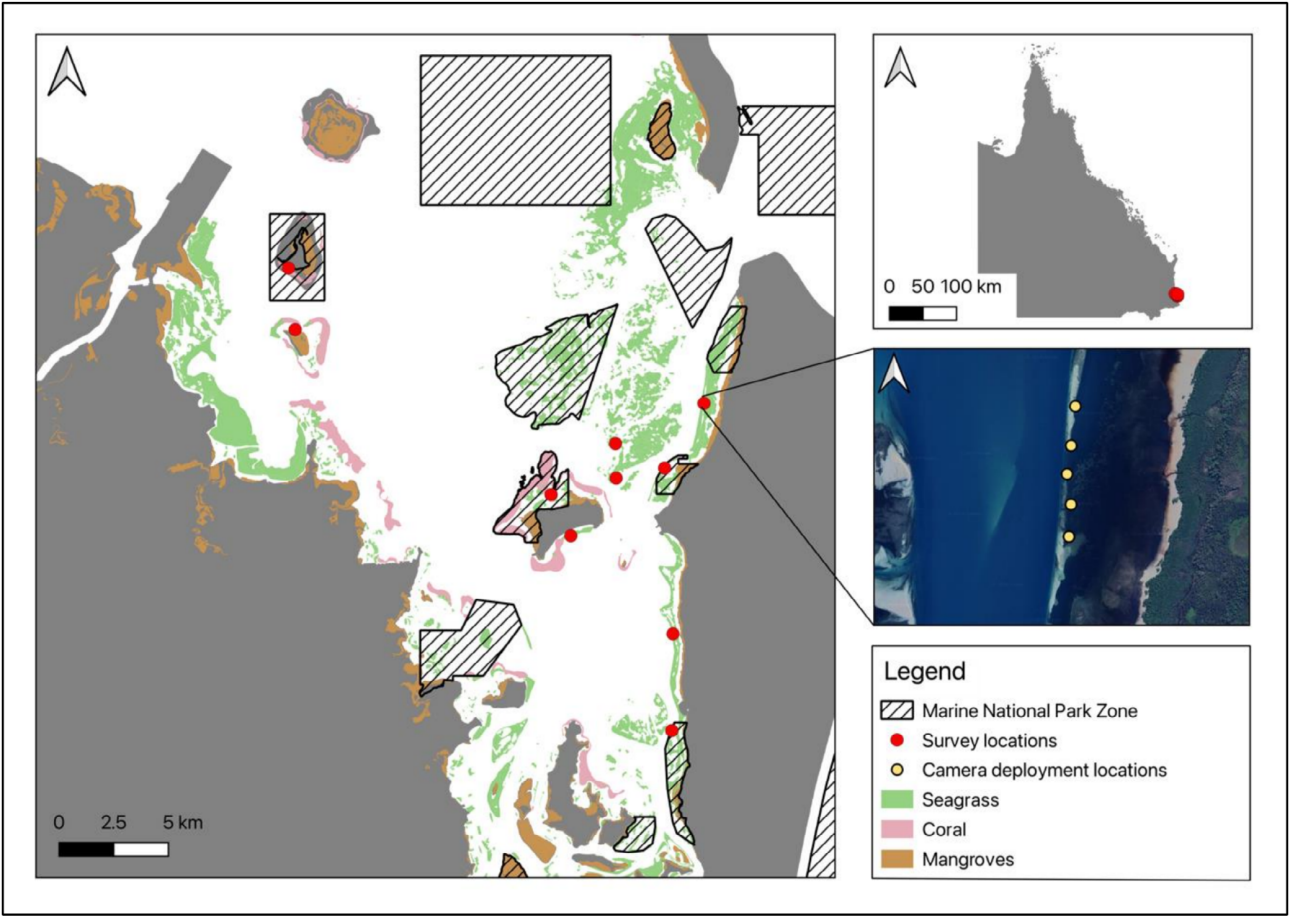
Map of the 10 survey locations in Moreton Bay, where BRUVS and RUVS were deployed. The top right inset depicts study site location in Queensland, Australia, middle inset shows the satellite imagery of the Wanga Wallen site with yellow dots indicating camera deployment locations.

We surveyed fish community structure, the rates of carnivory and herbivory, and the composition of seagrass meadows at 50 replicates across 10 seagrass meadow locations in Moreton Bay, Queensland, Australia (27°18′S; 153°17′ E) (Figure 1). Moreton Bay is a shallow (< 15 m depth) subtropical embayment spanning 1523 km^2^ that is enclosed eastward by three islands, Moreton Island and North and South Stradbroke Islands, and westward by Queensland's capital city, Brisbane, and adjacent urbanised regions. The south of Moreton Bay is dominated by mangrove‐lined estuaries and creeks from the western coastline that input nutrients and freshwater (Bennion et al. 2024). Moreton Bay is a multiple use marine park with four levels of activity zones: marine national park zones where all fishing and extractive processes are prohibited, conservation park zones which prohibit commercial fishing with some restrictions on recreational fishing, habitat protection zones where trawling is prohibited and general use zones where all forms of fishing are permitted within allowable catch limits. Surveyed locations were within four marine national park zones and six locations open to recreational fishing that were both near and far from the main coastline, the main estuary mouth, the open ocean and other important ecosystems, such as coral reefs and mangroves. Five of the six seagrass meadows outside of marine national park zones were located within habitat protection zones, with one seagrass meadow located within a conservation park zone. This seagrass meadow within the conservation park zone was chosen due to its proximity to coral reefs and moderate distance from the open ocean, which allowed for a more thorough test of the effects of connectivity. Given only one site was in a different zone, all unprotected sites were considered as the same level in the analysis.

### 2.2 Surveying Seagrass Fish Assemblages

Seagrass fish assemblages were surveyed with baited and unbaited remote underwater video systems (BRUVS and RUVS) (Henderson et al. 2017). Video deployments comprised a GoPro HD camera (GoPro Hero 10, recording in 4 k at 30 fps) centrally mounted to a 5 kg concrete paver that positioned imaging 10 cm above the seabed to provide a better field of view. A 1 m PVC pipe fixed to the concrete paver extended bait contents for BRUVS at an appropriate distance for species imaging (Langlois et al. 2020). BRUVS used 500 g of pilchards contained in a bait saver attached to the end of the PVC pipe. At each surveyed seagrass meadow (*n* = 10), five replicated BRUVS (*n* = 50) and five replicate RUVS (*n* = 50) were deployed; however, these two sampling methods were deployed on separate days to reduce fish sampling bias. To minimise the potential of imaging the same individual twice and maximise independence between replicates, each replicate was deployed at intervals of at least 200 m at each site (Langlois et al. 2020). To ensure species had sufficient water for transitioning through habitats and there was sufficient water visibility, all BRUVS and RUVS were deployed 2 h either side of the high tide (Goodridge Gaines et al. 2020). BRUVS were deployed for one hour (*n* = 50 h), while RUVS were deployed for 2 h (*n* = 100 h) to survey different components of the fish assemblage. RUVS were deployed for 2 h as they were unbaited and to maximise the opportunity of filming herbivory throughout the study. Species abundance and diversity were quantified from each video using the *MaxN* statistic, which is the maximum number of individuals of the same species present in a single video frame over the annotation period for each deployment, a conservative method ensuring individuals are not counted twice (Langlois et al. 2020).

### 2.3 Functional Assays

Functional assays were used to assess the rates of two ecological functions performed by fish; carnivory and herbivory. To quantify carnivory, 120 g (±20 g) of pilchards were attached to the outside of the BRUVS bait saver with a bait clip. Pilchards were reweighed with an electronic scale after the 1‐h deployment, with carnivory indexed as the net weight change of the pilchards (Goodridge Gaines et al. 2020; Henderson et al. 2020). To quantify herbivory, approximately 15 g (±5 g) of a common branching brown macroalgae (*Sargassum* spp.) present in Moreton Bay, and known to be grazed on by herbivorous fish (Gilby et al. 2017), was deployed at each replicate. *Sargassum* was dried in a salad spinner for 10 s, weighed with a Pesola Micro‐Line Precision Scale (precision ±0.3%) and attached to a terracotta tile (10 cm × 10 cm). Herbivory assays were deployed for 48 h and were imaged by RUVS for 2 h within the 48‐h period to identify herbivore assemblages and any species feeding on the *Sargassum* in that time. Following the assay retrieval, the remaining *Sargassum* was dried and reweighed by the same methods, and herbivory was indexed by the net weight change of the *Sargassum* (Gilby et al. 2017). During the assay video analysis, the intensity of fish carnivory rates was quantified by species removal of the assays; for example, if two species removed two pilchards each, both species would be attributed 50% of the carrion consumption (Müller et al. 2021; Mosman et al. 2023). This was repeated for herbivory; however, actual herbivory by fish was rarely filmed.

### 2.4 Classifying Seascape Variables and Habitat Composition

The effects of seascape spatial context known to influence fish assemblages and ecological functioning were investigated by classifying the distance to nearby habitats, the area of those habitats and the benthic composition of seagrass meadows (Henderson et al. 2019; Goodridge Gaines et al. 2020). Habitat connectivity promotes the abundance and diversity of fish communities (Olds et al. 2012) as fish require access to multiple habitats throughout different life stages for nurseries, feeding and reproduction. Consequently, the distance and area of nearby habitats which reflect these ecological needs were included. Analysis of seascape spatial variables was performed in QuantumGIS 3.38 and benthic habitat maps of seagrass meadows, mangrove forests and coral reefs were overlaid on AusMap satellite imagery with the coordinates of each survey site, the open ocean and main estuary mouth (Queensland Government 2015; Moustaka et al. 2024; QGIS Development Team 2024). The shortest possible path that did not overlap land between each surveyed replicate and the nearest coral reef, mangrove forest, estuary, and the open ocean was measured. The area of habitats nearby seagrass meadows was determined by applying a 500 m buffer to each surveyed replicate. 500 m buffers were selected to represent the relative maximum home ranges of fish species, thereby encompassing habitats species are utilising and transitioning through daily (Olds et al. 2012).

Benthic composition at each surveyed replicate was determined by identifying seagrass diversity and estimating seagrass cover, which was recorded as a percentage ranging from 0% to 100% in 5% increments. These estimates were identified from a still image obtained at the beginning of each RUVS video, with one image analysed per surveyed replicate. This approach provides a standardised and efficient method for characterising seagrass habitats and for comparison across surveyed replicates (Roelfsema et al. 2015; Tickler et al. 2017).

### 2.5 Calculating Functional Diversity

To calculate functional diversity, functional richness of both BRUVS and RUVS fish assemblages was quantified to describe the occupancy of trait space in seagrass meadows. Functional richness was indexed by the combination of three morphological and two ecological traits that characterise species functional roles (Henderson et al. 2024). In this study, maximum length was included due to this trait correlating with foraging capacity, type and vulnerability to predation risk, essential to ecosystem productivity (Nagelkerke and Rossberg 2014; Bellwood et al. 2019). Head length and eye diameter were included for their correlation with predation methods and predator type (Villéger et al. 2017). All species were categorised into one of the functional groups of detritivores, herbivores, omnivores, piscivores, zoobenthivores and zooplanktivores (Elliott et al. 2007). Integrating both ecological and morphological traits offers a more robust analysis of functional trait space and therefore, species capacity to respond to environmental disturbances (Schleuter et al. 2010; Nagelkerke and Rossberg 2014). Trait information for each fish species was sourced from FishBase, a global online database which contains trait information regarding morphology, feeding roles, and habitat types (Delgado et al. 2009). Functional richness was calculated using the *fundiv* package in R (Gagic et al. 2015).

### 2.6 Data Analysis

Initial analyses comparing the relationships between ecological functions and diversity metrics were performed in the R statistical environment (R Core Team 2024) using Generalised Linear Mixed Models (GLMMs) in the *glmmTMB* package (Brooks et al. 2017). The models tested correlations between fish functional richness and herbivory, carnivory, and seagrass diversity and cover. Analysis of variance (ANOVA) from the *car* package was used to determine the significance of predictors (Fox et al. 2013). To allow for relationships to reach an asymptote rather than being a straight line, models were limited to three knots using non‐linear splines in the *splines* package (Singh et al. 2019). Models on carnivory, herbivory, and seagrass diversity were modelled on the Tweedie error distribution, while seagrass cover was modelled on the beta error distribution as it is bound between 0 and 1, as seagrass cover was modelled as a proportion.

To determine relationships between functional richness and the ecological functions of herbivory and carnivory with the full suite of seascape and habitat metrics, Full Sub‐Set Generalised Additive Mixed Models (FSS GAMMs) were used in the package FSSgam in R (Fisher et al. 2018). Predictor variables included marine protected area status, distance to the open ocean, mangroves, coral reefs, main estuary mouth, and the area of seagrass, coral, and mangroves within a 500 m buffer. All models also included the random effect of seagrass meadow location. The carnivory, herbivory, and seagrass diversity models were calculated using the Tweedie error distribution, while functional richness measures and seagrass cover were calculated using the beta error distribution. Any models with AIC values within two of the best‐fit model were classified as equal in explanatory power.

Lastly, a FSS GAMM was calculated using the beta error distribution to test for correlations between ecosystem functioning and all seascape and habitat metrics. Ecosystem functioning was calculated by standardising each function and diversity metric between 0 and 1 by dividing all values by the maximum value across the dataset and then adding these together before again dividing all values by the maximum number. This resulted in a functioning value of between 0 and 1 for all replicates and equally weights rates of each of the different functional components measured.

## 3 Results

In this study, 56 fish species were identified across 50 surveyed replicates in 10 seagrass meadows in Moreton Bay, Queensland. A similar number of species were sampled by BRUVS (46 species) and RUVS (44 species), with 12 species unique to BRUVS and 10 species unique to RUVS. 17 fish species were identified performing carnivory during the 50 h of BRUVS imagery, with this being predominantly completed by the eastern striped trumpeter, *Pelates sexlineatus*, the eastern shovelnose ray, *Aptychotrema rostrata* , the smoothed stingray, *Dasyatis brevicaudata*, the silver toadfish, *Lagocephalus sceleratus* , and spinner shark, *Carcharhinus brevipinna* (Table 1). During the 100 h of RUVS imagery, very little herbivory was filmed, with the most common species recorded feeding being the omnivorous fan‐belly leather jacket *Monacanthus chinensis* .

**TABLE 1:** The proportion of carnivory completed by different species recorded on BRUVS across all replicates.

Common name	Species	Carnivory across all deployments (%)
Eastern striped trumpeter	*Pelates sexlineatus*	30%
Eastern shovelnose ray	*Aptychotrema rostrata*	14%
Smoothed stingray	*Dasyatis brevicaudata*	10%
Silver toadfish	*Lagocephalus sceleratus*	10%
Spinner shark	*Carcharhinus brevipinna*	8%
All remaining species		28%

### 3.1 Effects of Functional Richness on Ecological Functions in Seagrass Meadows

**FIGURE 2:**
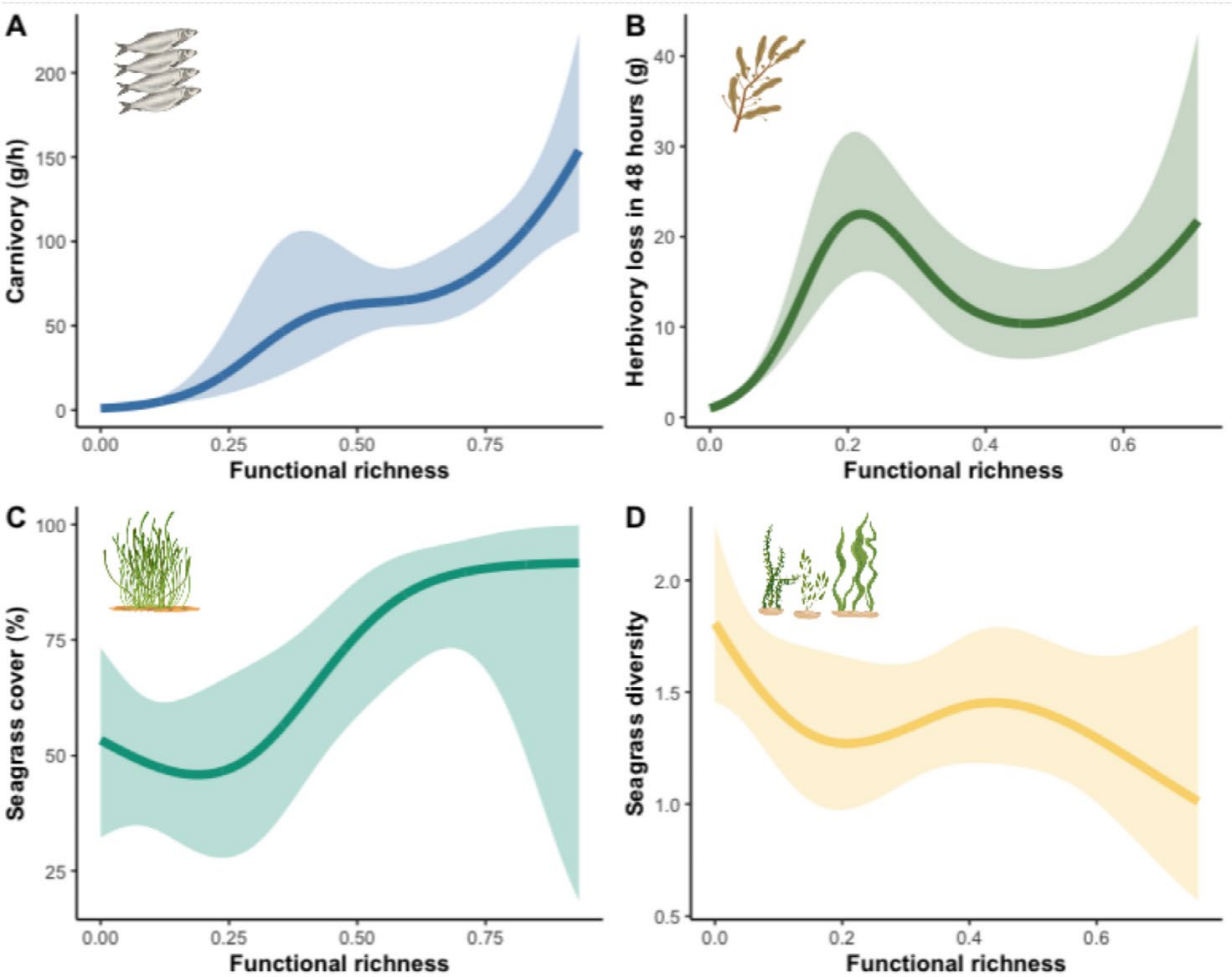
The functional richness of fish assemblages influences ecological functioning in seagrass meadows. Generalised linear mixed models highlighted significant changes in (A) the rates of carnivory, (B) the rates of herbivory and (C) seagrass cover (%); however, no significant effect was displayed for (D) the diversity of seagrass species with surveyed meadows.

The rate of carnivory (*X* ^2^ = 2401.3, *p* < 0.001) increased with increasing functional richness on BRUVS. Carnivory consistently increased with functional richness before reaching a maximum of approximately 150 g/h of carrion (Figure 2A and Table 2). The functional richness of all species imaged on RUVS initially correlated positively with the rate of herbivory (*X* ^2^ = 559, *p* < 0.001) to approximately 20 g/48 h of *Sargassum* and then plateaued with a moderate level of functional richness before displaying a subsequent rise with higher levels of functional richness (Figure 2B and Table 2). Seagrass cover (*X* ^2^ = 11.42, *p* = 0.009) significantly increased with functional richness. Seagrass meadow cover initially declines at lower levels of functional richness before steeply inclining at moderate levels of functional richness and then plateauing when functional richness was at its greatest (Figure 2C and Table 2). Seagrass diversity (*X* ^2^ = 5.61, *p* = 0.13) was not positively correlated with functional richness; seagrass diversity was highest when species functional diversity was lowest and consistently decreased with higher levels of functional richness (Figure 2D and Table 2).

**TABLE 2:** Generalised linear models testing for correlations between ecological functions and diversity variables, entire assemblage functional richness, species richness, and consumer assemblages.

Ecological function	Diversity variable	χ^2^	*p*	*R* ^2^
Carnivory				
	Functional richness	2401.3	**< 0.001**	0.42
	Species richness	3497	**< 0.001**	0.52
	Carnivore assemblage	2944.3	**< 0.001**	0.50
Herbivory				
	Functional richness	559	**< 0.001**	0.25
	Species richness	400.1	**< 0.001**	0.12
	Herbivore assemblage	84.1	**< 0.001**	0.49
Seagrass cover				
	Functional richness	11.42	**0.009**	0.22
	Species richness	1.96	0.58	0.49
Seagrass diversity				
	Functional richness	5.61	0.13	0.02
	Species richness	8.26	**0.023**	0.07

*Note:* Values in bold denote significance of *p* < 0.05.

Species richness had similar positive effects on the rates of carnivory and herbivory. However, opposing trends were identified in the relationship between species richness and seagrass diversity and cover in contrast with functional richness. Where seagrass diversity was significant (*X* ^2^ = 8.26, *p* = 0.023) and seagrass cover was insignificant (*X* ^2^ = 1.96, *p* = 0.58) (Figure S1 and Table 2). The rates of carnivory (*X* ^2^ = 3497, *p* < 0.001) in seagrass meadows were associated with increased species richness; carnivory steeply increased between 5 and 10 species before slightly increasing as species richness surpassed 10 species (Figure S1A and Table 2). The rates of herbivory (*X* ^2^ = 400.1, *p* < 0.001) displayed a non‐linear trend that was influenced by species richness; herbivory initially peaked at approximately four species before declining and subsequently rose again when species diversity was greatest (Figure S1B and Table 2). Seagrass diversity (*X* ^2^ = 8.26, *p* = 0.023) was greatest when species richness was lowest (Figure S1D and Table 2). Seagrass cover was insignificant and displayed a non‐linear trend, initially declining before increasing with species richness and displaying large confidence intervals (Figure S1D and Table 2). There was minimal variation between the functional richness of entire fish assemblages and the functional richness of consumer assemblages on the rates of carnivory (*X* ^2^ = 2944.3, *p* < 0.001) (Figure S2A and Table 2). Herbivory (*X* ^2^ = 84.1, *p* < 0.001) initially peaked at low levels of herbivore functional richness before gradually declining with large spanning confidence intervals (Figure S2B and Table 2).

### 3.2 Effects of Seascape Context on Ecological Functions in Seagrass Meadows

**FIGURE 3:**
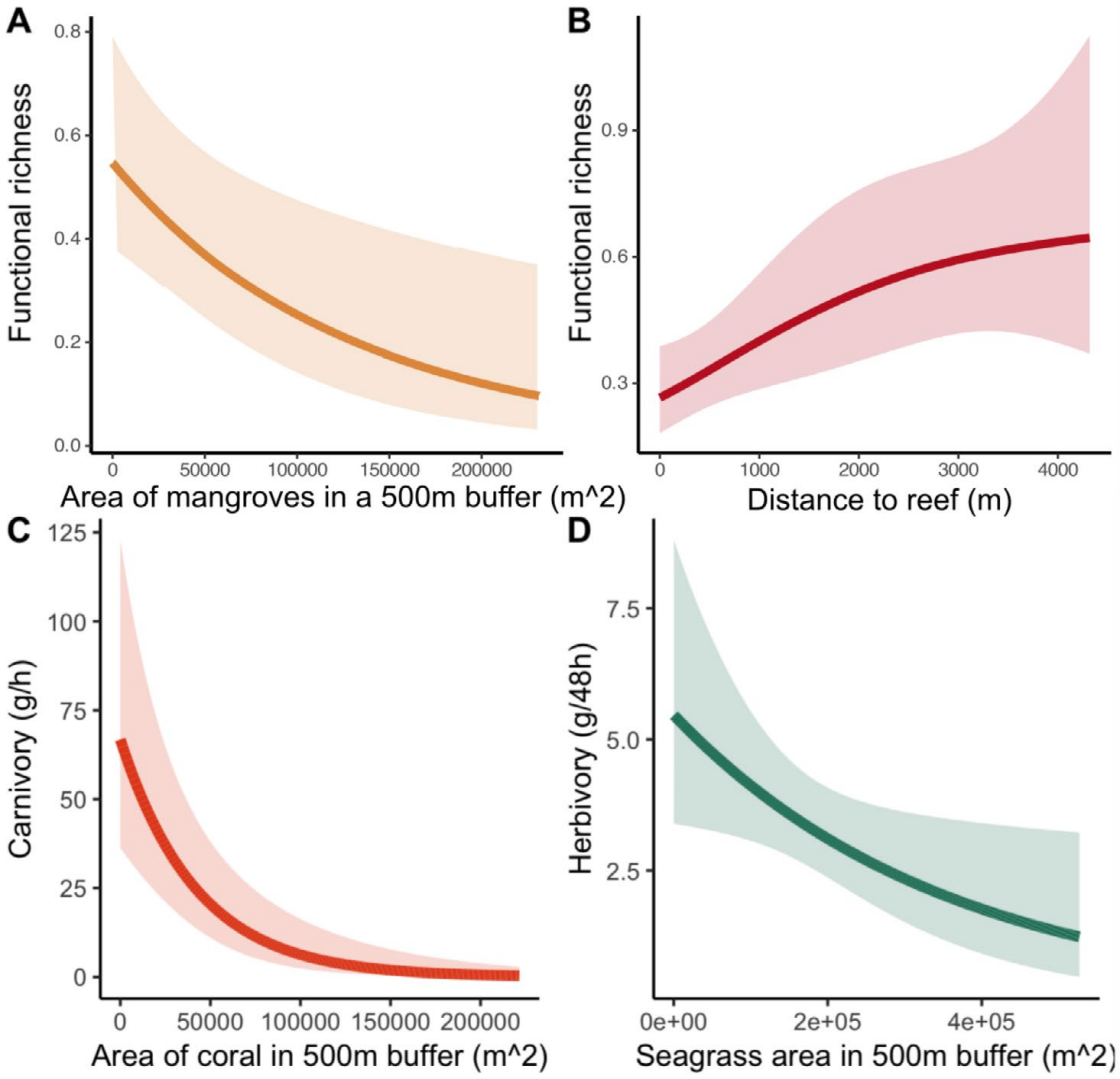
Seascape metrics drive ecological functioning in seagrass meadows; full subset generalised additive mixed models display significant relationships between functional richness recorded on BRUVS and (A) the area of mangroves and (B) the distance to coral reefs. The recorded ecological functions, carnivory and herbivory, correlated with (C) distance to the open ocean and (D) distance to coral reefs, respectively.

The functional richness of species identified on BRUVS, carnivory and herbivory were significantly influenced by spatial variation across the seascape, while RUVS functional richness, seagrass cover and diversity were not significantly related to any of the seascape metrics. The functional richness of fish assemblages identified by BRUVS in seagrass meadows was best explained by the joint effects of mangrove area within a 500 m buffer (*F* = 7.226, *p* = 0.01, *R* ^2^ = 0.53) and distance to coral reefs (*F* = 6.472, *p* = 0.018, *R* ^2^ = 0.53), where functional richness was greatest with less mangrove extent nearby and farther away from coral reefs (Figure 3A,B and Table 3). The area of coral in a 500 m buffer (*F* = 21.93, *p* < 0.001, *R* ^2^ = 0.76) best explained the rates of carnivory. The rates of carnivory also increased in closer proximity to coral reefs (Figure 3C and Table 3). Similarly, a single seascape metric characterised the rates of herbivory; the area of seagrass in a 500 m buffer (*F* = 5, *p* = 0.03, *R* ^2^ = 0.08) correlating with the removal of *Sargassum*, with herbivory being most prevalent in seagrass meadows with sparser seagrass (Figure 3D and Table 3).

**TABLE 3:** Full subset generalised additive mixed models assessing correlations between ecological functions and seascape metrics. All models include site as a random variable in the analysis, while those variables in bold are significant.

Ecological function	Best fit model	Deviance explained	*R* ^2^	# other models within 2AIC
Functional richness (RUVS)	Distance to ocean (*F* = 1.48, *p* = 0.23)	6.28%	0.07	4
Functional richness (BRUVS)	**Area of mangroves in a 500 m buffer (*F* = 7.226, *p* = 0.01)** + **Distance to reef (*F* = 6.472, *p* = 0.018)**	42.5%	0.53	9
Carnivory	**Area of coral in a 500 m buffer (*F* = 21.93, *p* < 0.001)**	70.8%	0.76	2
Herbivory	**Area of seagrass in a 500 m buffer (*F* = 5, *p* = 0.03)**	9.72%	0.08	0
Seagrass cover	Distance to ocean (*X* ^2^ = 3.05, *p* = 0.08) + Marine Park zoning (*Z* = −0.63, *p* = 0.52)	−16.6%	0.34	8
Seagrass diversity	Null			

### 3.3 Seascape Context on Entire Ecosystem Functioning

**FIGURE 4:**
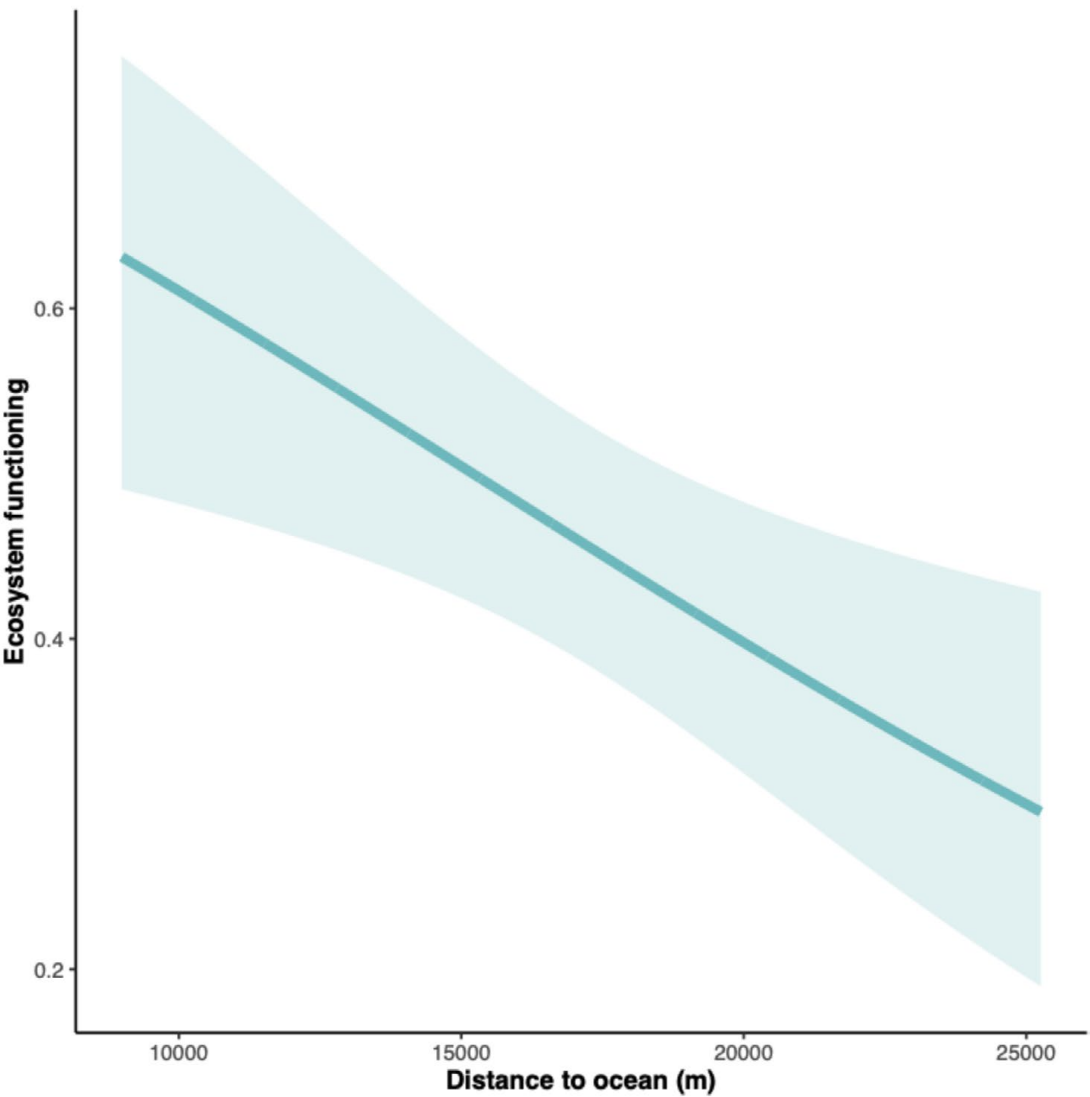
Full subset generalised additive mixed models show that proximity to the open ocean drives overall ecological functioning in seagrass meadows.

The distance to the open ocean (*X* ^2^ = 5.5, *p* = 0.019, *R* ^2^ = 0.67) was the sole predictor for entire ecosystem functioning and consequently was included in the best‐fit model without any other predictors (Table 4). Ecosystem functioning was greatest in closer proximity to the open ocean and consistently decreased with further distance (Figure 4).

**TABLE 4:** Full subset generalised additive mixed model assessing the correlation between overall ecological functioning and seascape metrics. The model includes site as a random variable in the analysis and variables in bold are significant.

Function	Best fit model	Deviance explained	*R* ^2^	# other models within 2AIC
Overall ecosystem functioning	**Distance to open ocean (X** ^**2**^ **= 5.5 *p* = 0.019)**	73%	0.67	9

## 4 Discussion

Ecological functions are important for maintaining the resilience, structure and functioning of ecosystems, and how these ecological functions relate to different components of faunal diversity or the composition of the seascape is key to understanding and managing the ecosystems in which they occur (Cadotte et al. 2011; Lefcheck and Duffy 2015; Villéger et al. 2017; deCastro‐Arrazola et al. 2023). However, it remains unclear how multiple ecological functions within an ecosystem such as seagrass meadows correlate with functional diversity (Mouillot et al. 2013; Lefcheck and Duffy 2015) and where they are maximised (Henderson et al. 2019). This study shows that the functional diversity of fishes is significantly correlated with carnivory and herbivory, therefore promoting multifunctionality in seagrass meadows. This study shows that there was consistency in the seascape context and connectivity variables that structured the rate and distribution of different components of functioning and the functional diversity of fish in seagrass meadows. The functional diversity of fish and the ecological functions of carnivory and herbivory correlated significantly with proximity to nearby reefs and the extent of seagrass, mangroves and coral reefs nearby. Finally, the overall ecosystem functioning within seagrass meadows was significantly modified by their position in the seascape relative to the open ocean, with locations closer having greater levels of functioning. These findings suggest that management strategies should prioritise the protection of seagrass meadows with the greatest levels of functional diversity to effectively conserve ecosystem multifunctionality in seagrass meadows (Schuldt et al. 2018; Goodridge Gaines et al. 2020).

Biological diversity has long been considered to be an important determinant of ecological functioning, yet recently, functional diversity has been shown to predict functioning better than such classic diversity measures (Gagic et al. 2015; Henderson et al. 2024). Here, functional richness significantly correlated positively with carnivory, herbivory and seagrass cover; however, species richness was a better predictor of the rate of carnivory within seagrass meadows. While it is of interest that species richness correlated with ecological functions in our study, this approach can be treated with caution as species richness does not rely on the identity of the species in the community and may miss dominant or invasive species (Lohbeck et al. 2016; Carvalho et al. 2022). However, for functional richness, species identity is key, with species identified by their traits (Lefcheck and Duffy 2015), which is instrumental in explaining the variance in ecological functioning (Cadotte et al. 2011) by indicating community trait occupancies, niche divergence, and therefore providing insights into the stability of ecosystem functioning (Schleuter et al. 2010; Villéger et al. 2017). Moreover, the functional diversity of both the carnivore and herbivore assemblages was significantly correlated with their respective functions in seagrass meadows. This is consistent with previous findings for functions such as herbivory, where performance varies depending on species traits (Scott et al. 2018), and while previously characterised by low redundancy in Moreton Bay (Gilby et al. 2017), is still a significant predictor of functioning. The link between functional diversity and ecological functions is central to conservation management (Harvey et al. 2017), but equally important is considering how seascape context shapes these relationships (Goodridge Gaines et al. 2022; Mosman et al. 2023).

Connectivity between ecosystems structures the composition of fish assemblages, the functions they deliver and, therefore, conservation management (Staveley et al. 2017; Maciel et al. 2024). The functional diversity of fish was significantly greater in seagrass meadows that had fewer mangroves nearby and were further from coral reefs, and that overall ecosystem functioning was greatest closer to the ocean. There were, however, no significant positive effects of conservation, which opposes the study's hypothesis and findings elsewhere assessing diversity and ecological function effects in marine reserves (Olds et al. 2012; Yabsley et al. 2016). This may suggest that the current network of reserves is not placed to provide the full benefits for multiple ecological functions or the functional diversity of fish (Henderson et al. 2019). The significant effects of ocean proximity on overall functioning may be linked with the biogeochemical characteristics of those habitats (Henley et al. 2020; Reinhard and Planavsky 2022). Seagrass meadows located near the open ocean receive an influx of nutrient‐poor water that often has increased light availability and promotes higher photosynthetic rates of seagrasses (Maxwell et al. 2014), in contrast to meadows closer to nutrient‐enriched estuaries (Gibbes et al. 2014; Saeck et al. 2019). These oceanic effects increase seagrass productivity and structural complexity which in turn promotes diversity (Jones et al. 2021). While it is well established that adjacent habitats generally support greater biodiversity due to an increase in resource availability (Berkström et al. 2020; Moustaka et al. 2024), the findings in this study revealed a greater level of functional diversity was associated with seagrass meadows situated further from coral reefs. There are two potential explanations for this: inter‐ecosystem resource competition and the degradation of nearby habitats. Previous studies have shown that seagrass‐associated fish increase in abundance at further distances from coral reefs (Dorenbosch et al. 2005), and that transitional spaces between seagrasses and nearby corals are characterised by lower fish densities due to lower structural complexity, which creates predation risk trade‐offs (Campbell et al. 2011). Alternatively, seagrass and coral reefs near the western fringes of Moreton Bay experience poorer water quality and lower light availability (Gibbes et al. 2014; Maxwell et al. 2014) with nutrient discharges from estuaries promoting algal biomass that degrade these habitats (Burkholder et al. 2007). Observations within this study align with these findings, particularly in the western region of the bay, where coral reefs near seagrass meadows exhibit higher algal biomass and lower coral cover, and seagrass meadows are less diverse and sparser in contrast to the eastern bay (Maxwell et al. 2014; Gilby et al. 2015; Henderson et al. 2017). The spatial arrangements and habitat conditions of seagrass meadows are important measures for determining the level of functional diversity in fish and the rates of ecological functions, and therefore understanding how these variables interact is key to managing functioning ecosystems (Tilman 2013; Yabsley et al. 2016).

Effectively functioning ecosystems support biodiversity and a myriad of ecosystem services (Harvey et al. 2017). Therefore, maintaining ecosystem functioning is integral to both ecological and human well‐being (Hector and Bagchi 2007; Nordlund et al. 2018). Here, the overall functioning in seagrass meadows is dependent on the positioning of seagrass meadows relative to the ocean, as highlighted by the significant relationship between overall ecosystem functioning and distance to ocean. Focusing conservation efforts in areas closer to the ocean should be a priority, but a deeper understanding of the specific characteristics of these adjacent seagrass meadows (Jones et al. 2021), and the fish communities they support (Staveley et al. 2017), will be essential to managing them, especially when subjected to less ideal conditions (Scott et al. 2018; Christianen et al. 2023). Seagrass meadows further from the open ocean have regular influxes of nutrient‐concentrated waters containing high levels of phosphorus and nitrogen that cause increased algae (Gibbes et al. 2014; Grinham et al. 2024). Moreton Bay receives 280,000 t of sediment per year delivered via the Brisbane River estuary, which has impacted 98% of the bay with fine sediment (Olley et al. 2006; Grinham et al. 2024). The effects of increased nutrient enrichment from catchments on coastal ecosystems are largely driven by urbanisation, industrial development and agricultural practices which are associated with the growing demands of the human population (Gibbes et al. 2014). Addressing these issues through targeted restoration efforts, such as the revegetation of bare estuarine banks, limiting direct livestock access to streams and protecting existing vegetation cover, is critical for mitigating the impacts of nutrient runoff (Olley et al. 2006; Gilby et al. 2016). These landscape management actions minimise eutrophication (Grinham et al. 2024), that could support increased functioning of Moreton Bays westward seagrass habitats.

This study has revealed that functional diversity significantly influenced multifunctionality and that both functional diversity and ecological functions were structured by the spatial context of the Moreton Bay seascape. Given these findings, it is recommended that prioritising the conservation of communities with increased levels of functional diversity in seagrass habitat management, for their strong influence on ecological functions. Moreover, it is critical to protect seagrass meadows closer to the open ocean, as this connectivity supports greater overall ecosystem functioning, with this potentially requiring changes to the current zoning within the marine park. The restoration of sediment and vegetation surrounding catchments to reduce nutrient output should be equally prioritised alongside the protection of seagrass meadows adjacent to the ocean. This study reinforces the need for functional diversity metrics that are integrated with spatially informed conservation strategies, ensuring the protection of critical ecological functions and the maintenance and delivery of ecosystem services such as coastal protection, biodiversity, nursery functioning and sediment filtering in seagrass meadows.

## Author Contributions

**Amarina L. James:** conceptualization (equal), data curation (equal), formal analysis (equal), investigation (equal), visualization (equal), writing – original draft (equal), writing – review and editing (equal). **Ben L. Gilby:** conceptualization (equal), supervision (equal), writing – review and editing (equal). **Andrew D. Olds:** conceptualization (equal), supervision (equal), writing – review and editing (equal). **Jesse D. Mosman:** conceptualization (equal), supervision (equal), writing – review and editing (equal). **Joshua T. Hill:** data curation (equal), investigation (equal), writing – review and editing (equal). **Christopher J. Henderson:** conceptualization (equal), formal analysis (equal), funding acquisition (equal), investigation (equal), methodology (equal), project administration (equal), supervision (equal), writing – review and editing (equal).

## Conflicts of Interest

The authors declare no conflicts of interest.

## Supporting information


**Figure S1:** Species richness influences ecological functioning, Species richness recorded on fish surveys modelled against (A) the rates of carnivory, (B) the rates of herbivory, (C) seagrass cover (%) and (D) the diversity of seagrass species in surveyed meadows.
**Figure S2:** Functional richness of consumer assemblages modelled against the two animal driven ecological functions, (A) the rates of carnivory and (B) the rates of herbivory.

## Data Availability

All the required data are uploaded as Data S1.
