# Supplementary material for: Functionally Rich Fish Assemblages Support Greater Rates of Multiple Ecological Functions in Seagrass Meadows
Source: Ecol Evol. 2026 Jan 30;16(2):e73011. doi: 10.1002/ece3.73011 (PMC12856372; doi:10.1002/ece3.73011)
Supplement: Supplementary file 1 — Figure S1: Species richness influences ecological functioning, Species richness recorded on fish surveys modelled against (A) the rates of carnivory, (B) the rates of herbivory, (C) seagrass cover (%) and (D) the diversity of seagrass species in surveyed meadows. Figure S2: Functional richness of consumer assemblages modelled against the two animal driven ecological functions, (A) the rates of carnivory and (B) the rates of herbivory. [file ECE3-16-e73011-s001.docx]

**Supplementary Information**

**
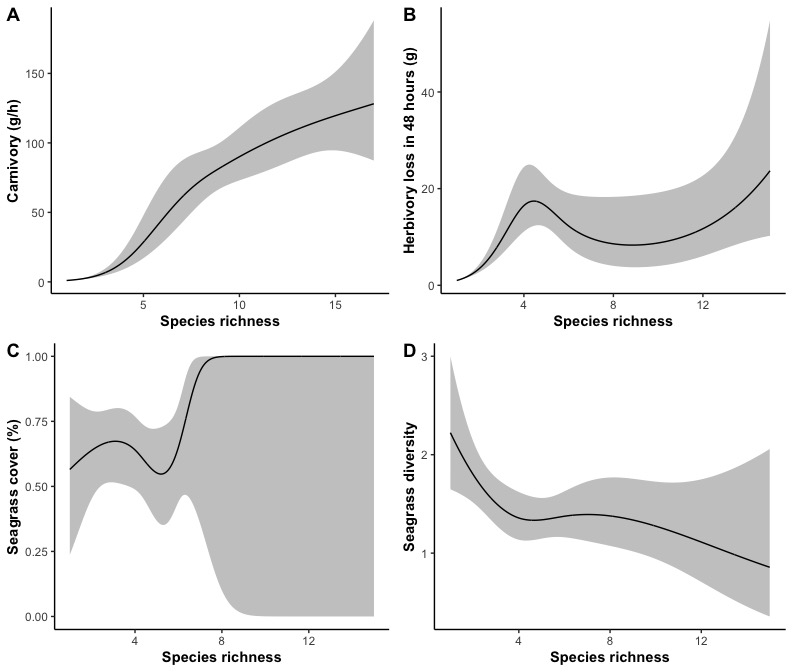
**

Figure S1: Species richness influences ecological functioning, Species richness recorded on fish surveys modelled against (**A**) the rates of carnivory, (**B**) the rates of herbivory, (**C**) seagrass cover (%) and (**D**) the diversity of seagrass species in surveyed meadows.

**
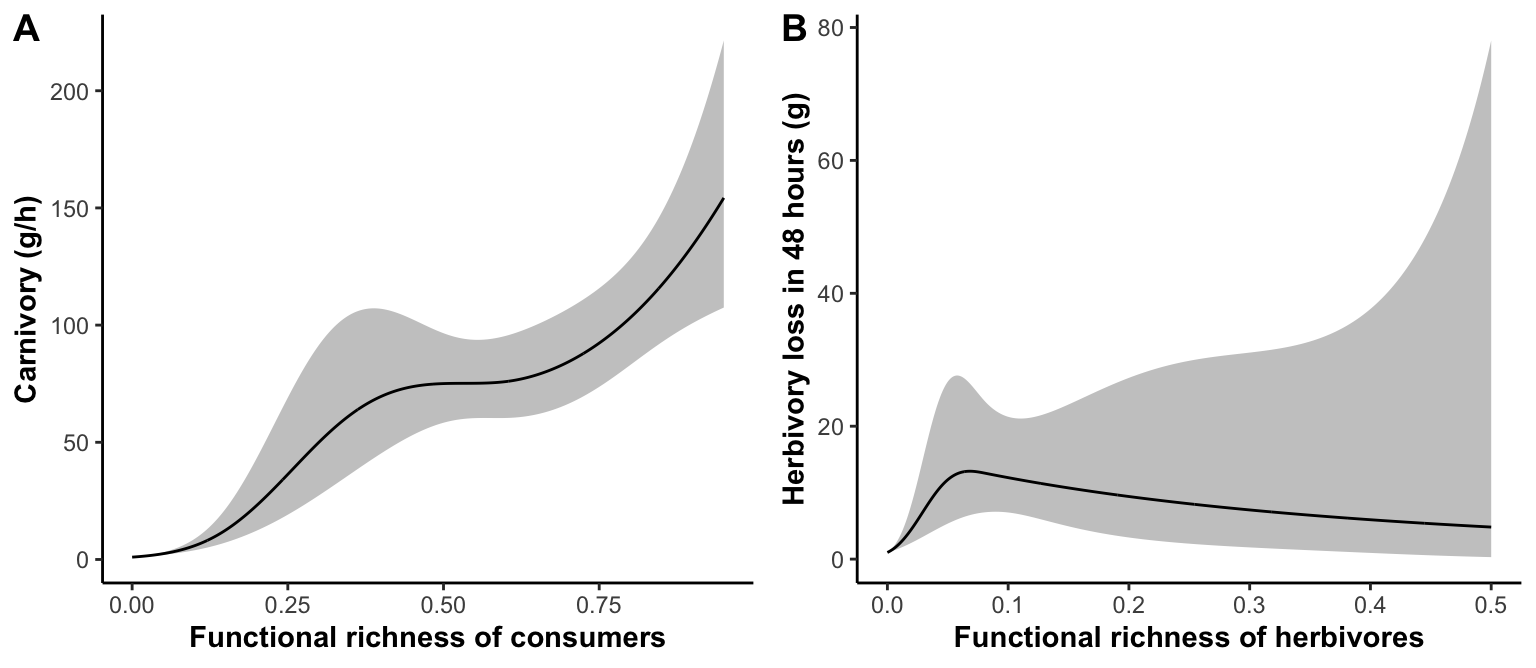
**

Figure S2: Functional richness of consumer assemblages modelled against the two animal driven ecological functions, (**A)** the rates of carnivory and (**B)** the rates of herbivory.
